# Effects of Outdoor Therapy on Delirium in Patients With Prolonged Intensive Care Unit Stays: A Single‐Centre Retrospective Study

**DOI:** 10.1111/nicc.70263

**Published:** 2025-11-19

**Authors:** Daichi Tsukakoshi, Hitoshi Mutai, Shuhei Yamamoto, Masaaki Sato, Keisuke Furuhashi, Toshinori Nakamura, Takashi Ichiyama, Hiroshi Imamura, Hiroshi Horiuchi

**Affiliations:** ^1^ Department of Rehabilitation Shinshu University Hospital Matsumoto Japan; ^2^ Department of Health Science, Graduate School of Medicine, Science and Technology Shinshu University Matsumoto Japan; ^3^ Department of Occupational Therapy, School of Health Sciences Shinshu University Matsumoto Japan; ^4^ Department of Psychiatry Shinshu University School of Medicine Matsumoto Japan; ^5^ Department of Intensive Care Unit Shinshu University Hospital Matsumoto Japan

**Keywords:** delirium, early mobilisation, intensive care unit, non‐pharmacological intervention, outdoor therapy

## Abstract

**Background:**

Delirium commonly occurs during prolonged intensive care unit (ICU) stays, yet the clinical benefit of escorted outdoor exposure for critically ill adults remains uncertain.

**Aim:**

The aim of this study was to determine whether supervised outdoor therapy is associated with reduced delirium severity among adults with ICU stays ≥ 7 days.

**Study Design:**

We performed a single‐centre, retrospective cohort study in Japan, including adults with an ICU length of stay ≥ 7 days from 1 January 2019 to 31 December 2022. Delirium was assessed twice‐daily using the Intensive Care Delirium Screening Checklist (ICDSC). We evaluated (1) within‐patient change from 16:00 on the day before to 16:00 on the day of the first outdoor‐therapy session; (2) ICDSC score at ICU discharge comparing patients who did vs. did not receive outdoor therapy using multiple imputation integrated with propensity‐score matching; and (3) dose–response using multivariable linear regression with session count, adjusting for prespecified covariates including the highest ICDSC score and psychotropics prescribed through the first session.

**Results:**

Among patients (*n* = 391) receiving outdoor therapy, the median ICDSC score decreased from 4 to 2 on the day of the first session (*p* < 0.001). After propensity‐score matching, the outdoor‐therapy group had lower ICDSC scores at ICU discharge than matched controls (median: 3.0 [IQR 1.0–5.0] vs. 4.0 [2.0–6.0]; *p* = 0.013; Cliff's *δ* = −0.329). In the adjusted regression, a greater number of outdoor sessions was associated with a lower discharge ICDSC score (standardised *β* = −0.113; *p* = 0.025).

**Conclusions:**

Supervised outdoor therapy was associated with lower delirium severity in adults with prolonged ICU stays. These findings support the integration of outdoor exposure within multimodal delirium management while prospective multicentre studies define standardised protocols and optimal dosing.

**Relevance to Clinical Practice:**

Incorporating brief, supervised outdoor sessions into multidisciplinary ICU workflows may provide a practical, non‐pharmacological adjunct for delirium care, pending confirmation in protocolised prospective trials.

**Trial Registration:** University Hospital Medical Information Network Clinical Trial Registry (UMIN‐CTR) (ID: UMIN000049057; registered on 1 October 2022; https://center6.umin.ac.jp/cgi‐open‐bin/ctr_e/ctr_view.cgi?recptno=R000055872)

**Impact Statements**

- What is known about the topic
  ○ Early mobilisation is recommended in multiple consensus statements and guidelines to mitigate ICU delirium.
  ○ Structured outdoor exposure has been found to reduce agitation in dementia and mental‐health settings, but evidence in critical care is limited.
- What this paper adds
  ○ Outdoor therapy was associated with same‐day improvement and lower ICU‐discharge ICDSC scores in adults with prolonged ICU stays.
  ○ Our findings highlight outdoor therapy as a feasible, non‐pharmacological adjunct to existing delirium bundles, warranting the evaluation of protocolised outdoor therapy for establishing the optimal dose and candidate selection.

## 1 Introduction

Delirium during critical illness is common and clinically consequential, being associated with prolonged hospitalisation, postoperative complications and mortality [1], long‐term cognitive decline [2] and increased costs [3]; its prevention in the intensive care unit (ICU) is therefore a priority. Contemporary practice guidelines emphasise non‐pharmacological strategies—reducing modifiable risks, supporting cognition and sleep, promoting early mobilisation and addressing sensory needs [4]. In particular, early mobilisation has been linked to improved functional capacity [5], reductions in cognitive symptoms including delirium [6, 7, 8, 9, 10], shorter ICU length of stay [11, 12] and improved long‐term outcomes [13] and is consequently endorsed by expert consensus and guidelines [4, 14, 15].

Supervised outdoor therapy—escorting of ICU patients outdoors to experience natural sensory stimuli—has been proposed as a pragmatic extension of early mobilisation [16, 17, 18]. Its rationale draws on the biophilia hypothesis and observations from other care settings: Outdoor group activities can improve sleep, and exposure to natural elements (e.g., plants, daylight, and nature views) can mitigate agitation and cognitive stress [19, 20, 21, 22, 23, 24, 25]. Prior studies have reported associations between patient‐room nature views and clinical outcomes and costs [26, 27], based on guidance issued by the UK Intensive Care Society, and have described the prevalence, content and safety protocols of such initiatives [16, 17, 18, 28]. Nevertheless, despite this growing interest, the clinical effectiveness of outdoor therapy for ICU patients has not been directly evaluated.

Accordingly, we investigated whether supervised outdoor therapy is associated with reduced delirium severity in adults with prolonged ICU stays. We examined the immediate, within‐patient change on the day of the first outdoor session and between‐group differences at ICU discharge, with adjustment for confounding, hypothesising that outdoor exposure—and a greater number of sessions—would be associated with reduced delirium severity. We focussed on adults with an ICU length of stay ≥ 7 days because extended critical illness increases the risk and persistence of delirium and affords sufficient opportunity to deliver and evaluate supervised outdoor exposure.

## 2 Aim and Objectives

We aimed to determine whether supervised outdoor therapy is associated with reduced delirium severity among adults with prolonged ICU stays (≥ 7 days). The primary objective was to compare Intensive Care Delirium Screening Checklist (ICDSC) scores at ICU discharge between patients who did and did not receive outdoor therapy using multiple imputation integrated with propensity‐score matching. The secondary objectives were as follows: (i) to quantify within‐patient change in ICDSC from 16:00 on the day before to 16:00 on the day of the first outdoor session, and (ii) to determine whether a dose–response relationship exists between the number of outdoor sessions and discharge ICDSC score, using multivariable regression adjusted for prespecified confounders, including illness severity and psychotropic exposure.

## 3 Design and Methods

### 3.1 Study Design and Participants

We conducted a single‐centre, retrospective cohort study in Matsumoto, Nagano, Japan, reported in accordance with the Strengthening the Reporting of Observational Studies in Epidemiology (STROBE) guidelines [29]. We included all critically ill medical and surgical adults with an ICU length of stay ≥ 7 days between 1 January 2019 and 31 December 2023. Inclusion was restricted to ≥ 7 days to ensure persistent delirium risk and sufficient opportunity for delivery of outdoor therapy and pre‐ and post‐exposure ICDSC assessment; patients with shorter stays were unlikely to receive the intervention or permit meaningful evaluation. Patients were excluded if they were maintained on absolute bed rest, died during the ICU stay or had more than one ICU admission during the index hospitalisation; deaths were excluded because the primary outcome—ICDSC at ICU discharge—could not be ascertained.

### 3.2 Data Sources and Variables

The following data were extracted from electronic medical records: pre‐ICU status (age, sex, body‐mass index [BMI], Brinkman index, history of stroke, haemoglobin and Clinical Dementia Rating [CDR]), comorbidity (Charlson Comorbidity Index [CCI]) [30], severity at ICU admission (Acute Physiology and Chronic Health Evaluation II [APACHE II]) [31], organ dysfunction at ICU admission (Sequential Organ Failure Assessment [SOFA]) [32] and ICU treatments (duration of mechanical ventilation, use of continuous renal replacement therapy [CRRT], intra‐aortic balloon pump [IABP], percutaneous cardiopulmonary support [PCPS] and ICU length of stay).

### 3.3 Exposure: Outdoor Therapy

Outdoor therapy was defined as exiting the ICU to an outdoor area—by bed, wheelchair or ambulation—to observe scenery and experience ambient temperature, wind, sounds, smells and seasonal stimuli. We recorded whether therapy occurred, the number of sessions and transport mode. No protocolised minimum or maximum duration was set; session length was pragmatically determined by clinical status, therapy goals, staffing and weather and was not systematically recorded. The therapy was implemented when the perceived benefits outweighed the risks after a multidisciplinary review of the patient's physical and mental status, weather, staffing and related factors at daily morning rounds attended by intensivists, nurses, clinical engineers and occupational/physical therapists. The ICU was located on the second floor; therefore, the patients were transported to the first floor by elevator and then assisted outside near one of the hospital entrance gates (Figure S1). Because session duration was not captured in a systematic field, dose was operationalised as the number of outdoor sessions per patient.

### 3.4 Assessment of Delirium and Outcomes

Bedside nurses assessed the patients for delirium twice‐daily using the ICDSC at 04:00 and 16:00 from ICU admission to discharge; ICDSC scores ≥ 4 indicated delirium [33]. To evaluate the immediate effects, we compared ICDSC values at 16:00 on the day before the first outdoor session with those reported at 16:00 on the day of that session. In the between‐group and dose–response analyses, we recorded the highest in‐ICU and ICU‐discharge ICDSC scores.

Delirium persisting at ICU discharge is prognostic: It is associated with increased mortality, prolonged hospitalisation, discharge to institutional care and increased costs; it also predicts cognitive decline after critical illness [1, 2, 3]. Accordingly, we designated the discharge ICDSC as the primary outcome. Immediate post‐session values were reserved for use as a key secondary outcome because delirium fluctuates—rendering near‐intervention readings labile—and short‐term sedation or agitation can distort assessment. A standardised discharge assessment therefore provides a stable, clinically interpretable anchor. Evidence that early rehabilitation improves outcomes in critically ill populations underscores the clinical relevance of achieving milder delirium by the time of discharge for promoting ward‐based therapy and recovery [5, 9, 11, 12].

### 3.5 Medication Covariates

We recorded all psychotropic medications prescribed for insomnia or delirium from ICU admission to the first outdoor session (antipsychotics: aripiprazole, asenapine, olanzapine, perospirone, quetiapine and risperidone; antidepressants: milnacipran, mirtazapine and trazodone; insomnia agents: benzodiazepine‐receptor‐agonist anxiolytics/hypnotics and non‐benzodiazepine‐receptor‐agonist hypnotics, as well as lemborexant, ramelteon and suvorexant). To address potential confounding in the within‐patient comparison, we verified that no initiation or discontinuation occurred within 72 h before the first outdoor session, considering time to steady state and washout.

### 3.6 Statistical Analysis

Baseline characteristics are summarised as median [interquartile range] or number (percentage). Between‐group comparisons were descriptive and two‐sided. For continuous variables, we used the Wilcoxon rank–sum test and reported the test statistic (W) alongside an effect‐size estimate given by Cliff's *δ* with 95% confidence intervals. For binary variables, we used Fisher's exact test (two‐sided) and reported odds ratios (ORs) with 95% confidence intervals. For sparse cells yielding undefined ORs, values were denoted NE (not estimable) while retaining the Fisher's exact *p* value.

To compare ICDSC scores at ICU discharge between the groups while accounting for confounding, we used multiple imputation combined with propensity‐score (PS) matching. The missing‐data pattern was examined visually and using Little's test of missing completely at random (MCAR). Imputation was performed using Multivariate Imputation by Chained Equations (mice) with predictive mean matching (m = 20; max iterations = 50; seed = 1234). We then applied the ‘within’ approach to integrate PS methods across the imputed datasets [34]. The PS was estimated by logistic regression with receipt of outdoor therapy as the dependent variable and the following prespecified covariates: age, sex, Brinkman index, body mass index (BMI), Charlson Comorbidity Index (CCI), Clinical Dementia Rating (CDR), Acute Physiology and Chronic Health Evaluation II (APACHE II), Sequential Organ Failure Assessment (SOFA), emergency surgery, continuous renal replacement therapy (CRRT), intra‐aortic balloon pump (IABP), percutaneous cardiopulmonary support (PCPS), all psychotropics prescribed for delirium up to the first outdoor session and highest in‐ICU ICDSC score. We performed 1:1 nearest‐neighbour matching with a calliper of 0.2 (PS standard deviation); post‐match comparisons used the Wilcoxon rank–sum test.

For dose–response analyses, we modelled ICU‐discharge ICDSC as the outcome in simple and multivariable linear regressions with receipt and number of outdoor sessions as key predictors. The adjusted model (fitted after multiple imputation) included the prespecified confounders listed above [35, 36, 37, 38], medications for delirium up to the first outdoor session and the highest ICDSC score. Two‐sided *p* < 0.05 was considered statistically significant. Analyses were conducted in R, version 4.4.1 (R Foundation for Statistical Computing).

### 3.7 Ethics and Oversight

The study complied with the Declaration of Helsinki and was approved by the Ethics Committee of Shinshu University Hospital (approval No. 5624; approved on 14 September 2022). The requirement for written informed consent was waived in favour of an opt‐out process via institutional website disclosure. The study was registered with the University Hospital Medical Information Network Clinical Trial Registry (UMIN‐CTR) (Unique ID issued by UMIN000049057; registered on 1 October 2022; https://center6.umin.ac.jp/cgi‐open‐bin/ctr_e/ctr_view.cgi?recptno=R000055872 withheld for peer review).

## 4 Results

### 4.1 Participants

From 1 January 2019 through 31 December 2022, 478 patients remained in the ICU for ≥ 7 days. Of these, 87 were excluded (no rehabilitation, *n* = 4; died in the ICU, *n* = 66; ICU readmission, *n* = 17), resulting in 391 in the final analysis (outdoor therapy group, *n* = 59; non‐outdoor therapy group, *n* = 332) (Figure S2).

At baseline, patients in the outdoor‐therapy group were older, had longer ICU length of stay and were more likely to receive medications for delirium (notably asenapine, ramelteon and risperidone). Delirium during the ICU stay and the highest ICDSC score were more frequent/higher in the outdoor‐therapy group; the unadjusted ICDSC at ICU discharge did not differ between the groups (Table 1).

**TABLE 1:** Baseline characteristics of patients.

	Outdoor therapy group *n* = 59	Non‐outdoor therapy group *n* = 332	Test and statistic	Effect size (95% CI)	*p* value
Pre‐ICU admission status					
Age—year	76.0 [62.0, 81.0]	68.0 [50.0, 77.0]	*W* = 7252^a^	*δ* = 0.26 (0.09–0.41)	0.001
Female sex—no. (%)	37 (62.7)	210 (63.3)	Fisher's exact^b^	OR = 0.98 (0.53–1.82)	1.000
BMI (kg/m^2^)	22.7 [21.0, 26.4]	23.1 [20.4, 26.2]	*W* = 9734^a^	*δ* = 0.01 (−0.15–0.16)	0.940
Brinkman index	0.0 [0.0, 400.0]	0.0 [0.0, 475.0]	*W* = 9293^a^	*δ* = −0.04 (−0.19–0.11)	0.607
CCI	4.0 [1.0, 6.0]	4.0 [2.0, 7.0]	*W* = 10 066^a^	*δ* = −0.03 (−0.18–0.13)	0.732
CDR	0.0 [0.0, 0.0]	0.0 [0.0, 0.0]	*W* = 9586^a^	*δ* = 0.02 (−0.06–0.11)	0.595
During ICU stay status					
Emergency operation—no. (%)	33 (55.9)	136 (40.9)	Fisher's exact^b^	OR = 1.83 (1.01–3.34)	0.045
APACHE II score	33.0 [25.5, 38.0]	32.0 [25.0, 36.0]	*W* = 9182^a^	*δ* = 0.06 (−0.10–0.22)	0.418
SOFA	10.0 [7.5, 13.0]	10.0 [7.0, 12.0]	*W* = 9149^a^	*δ* = 0.07 (−0.10–0.22)	0.418
Mechanically ventilated—day	3.0 [1.0, 16.8]	5.0 [0.0, 10.0]	*W* = 8142^a^	*δ* = 0.04 (−0.14–0.21)	0.664
ICU LOS—day	20.0 [12.0, 41.0]	12.0 [9.0, 19.0]	*W* = 5699^a^	*δ* = 0.42 (0.25–0.56)	< 0.001
CRRT—no. (%)	17 (28.8)	47 (14.2)	Fisher's exact^b^	OR = 2.45 (1.20–4.83)	0.008
IABP—no. (%)	7 (11.9)	37 (11.1)	Fisher's exact^b^	OR = 1.07 (0.38–2.61)	0.825
PCPS—no. (%)	11 (18.6)	55 (16.6)	Fisher's exact^b^	OR = 1.15 (0.51–2.43)	0.707
Pre‐session psychotropics (delirium/insomnia)—no. (%)					
Aripiprazole	1 (1.7)	1 (0.3)	Fisher's exact^b^	OR = 5.67 (0.07–447.55)	0.279
Asenapine	13 (22.0)	21 (6.3)	Fisher's exact^b^	OR = 4.16 (1.79–9.42)	< 0.001
Olanzapine	3 (5.1)	11 (3.3)	Fisher's exact^b^	OR = 1.56 (0.27–6.17)	0.453
Perospirone	2 (3.4)	10 (3.0)	Fisher's exact^b^	OR = 1.13 (0.12–5.50)	0.700
Quetiapine	10 (16.9)	43 (13.0)	Fisher's exact^b^	OR = 1.37 (0.58–3.01)	0.411
Risperidone	18 (30.5)	39 (11.7)	Fisher's exact^b^	OR = 3.29 (1.61–6.55)	0.001
Milnacipran	0 (0.0)	1 (0.3)	Fisher's exact^b^	NE	1.000
Mirtazapine	0 (0.0)	4 (1.2)	Fisher's exact^b^	NE	1.000
Trazodone	5 (8.5)	15 (4.5)	Fisher's exact^b^	OR = 1.95 (0.53–5.96)	0.203
BZRA/anxiolytics	2 (3.4)	11 (3.3)	Fisher's exact^b^	OR = 1.02 (0.11–4.88)	1.000
BZRA/hypnotic	3 (5.1)	8 (2.4)	Fisher's exact^b^	OR = 2.16 (0.36–9.37)	0.222
Non‐BZRA/hypnotic	4 (6.8)	37 (11.1)	Fisher's exact^b^	OR = 0.58 (0.14–1.71)	0.487
Lemborexant	31 (52.5)	150 (45.2)	Fisher's exact^b^	OR = 1.34 (0.74–2.44)	0.323
Ramelteon	41 (69.5)	157 (47.3)	Fisher's exact^b^	OR = 2.53 (1.36–4.89)	0.002
Suvorexant	8 (13.6)	62 (18.7)	Fisher's exact^b^	OR = 0.68 (0.27–1.55)	0.461
ICU delirium status					
During ICU stay—no. (%)	56 (94.9)	270 (81.6)	Fisher's exact^b^	OR = 4.21 (1.30–21.70)	0.008
At ICU discharge—no. (%)	26 (44.1)	115 (34.7)	Fisher's exact^b^	OR = 1.48 (0.81–2.69)	0.187
Highest ICDSC score	7.0 [6.0, 8.0]	6.0 [4.0, 7.0]	*W* = 6879^a^	*δ* = 0.30 (0.16–0.43)	< 0.001
ICDSC score at ICU discharge	3.0 [1.0, 5.0]	2.0 [1.0, 5.0]	*W* = 9547^a^	*δ* = 0.03 (−0.14–0.19)	0.755

*Note:* Values are presented as median [interquartile range], no (%), or mean (standard deviation).

Abbreviations: APACHE, acute physiology and chronic health evaluation; BMI, body mass index; BZRA, benzodiazepine receptor agonists; CCI, Charlson comorbidity index; CDR, clinical dementia rating; CRRT, continuous renal replacement therapy; IABP, intra‐aortic balloon pumping; ICU, intensive care unit; ICDSC, intensive care delirium screening checklist; LOS, length of stay; NE, not estimable; OR, odds ratio; PCPS, percutaneous cardiopulmonary support.

^a^ Wilcoxon rank–sum test for continuous variables (reported with W); effect size reported as Cliff's *δ* with 95% confidence intervals.

^b^ Fisher's exact test (two‐sided) for binary variables; effect size reported as odds ratio (OR) with 95% confidence intervals. NE denotes not estimable (e.g., zero cells). Values are presented as median [interquartile range] or number (percentage).

### 4.2 Delivery of Outdoor Therapy

Among the recipients (*N* = 59), the median time from ICU admission to the first session was 22 days (interquartile range, 15–43). Most patients had received one (69.4%) or two (18.6%) sessions; 13.5% were transported by bed, 83.0% by wheelchair and 3.3% walked (Table S1). All outdoor‐therapy sessions were undertaken after the highest ICDSC score had already been recorded.

### 4.3 Immediate Within‐Patient Change on the First Day

**FIGURE 1:**
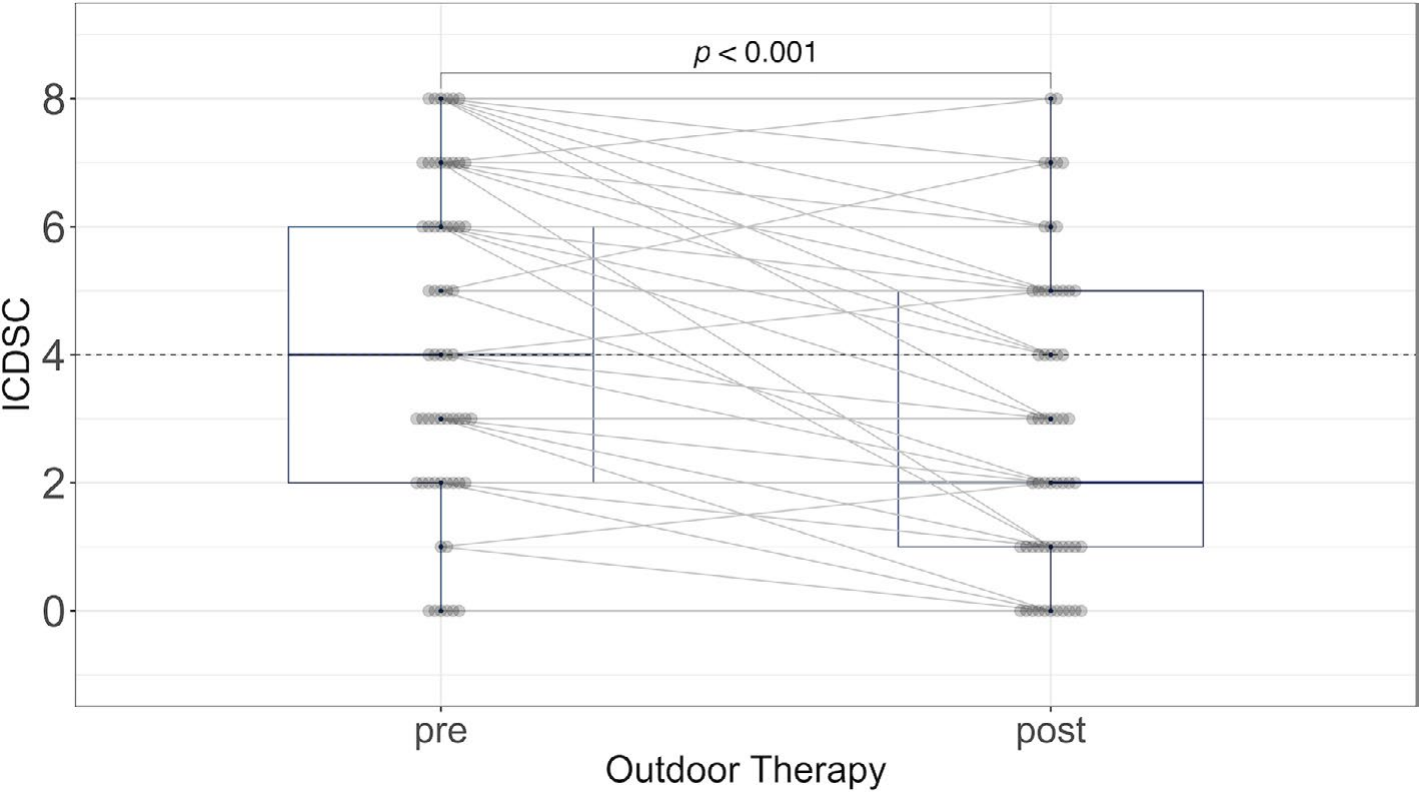
Comparison of intensive care delirium screening checklist scores before and after outdoor therapy. ICDSC, intensive care delirium screening checklist.

Per the protocol, immediate change was defined as a change in the ICDSC score at 16:00 on the day before versus the score at 16:00 on the day of the first session. The median ICDSC score decreased from 4.0 to 2.0 (*p* < 0.001) (Figure 1). No delirium‐related medications were initiated or discontinued within the 72 h preceding the session.

### 4.4 Propensity‐Score–Matched Comparison at ICU Discharge

Missingness was limited (Brinkman index 5.12%; ventilator duration 6.14%), consistent with MCAR (Little's test *p* = 0.271); multiple imputation was applied (Figure S3). Propensity‐score matching yielded 57 patients per group with standardised mean differences < 0.1 for all covariates (Figures S4 and S5). In the matched cohort, the ICDSC score at ICU discharge was lower in the outdoor‐therapy group (median 3.0 [1.0–5.0]) than in the matched control group (4.0 [2.0–6.0]; *p* = 0.013), with Cliff's *δ* = −0.329 (Table 2).

**TABLE 2:** Propensity score analysis for outdoor therapy and intensive care unit delirium.

	Before propensity‐score matching				After propensity‐score matching^a^			
	Outdoor therapy *n* = 59	Non‐outdoor therapy *n* = 332	Cliff's *δ* ^b^	*p*	Outdoor therapy *n* = 57	Non‐outdoor therapy *n* = 57	Cliff's *δ* ^b^	*p*
ICDSC score								
At ICU discharge	3.0 [1.0, 5.0]	2.0 [1.0, 5.0]	0.025 (−0.135, 0.185)	0.755	3.0 [1.0, 5.0]	4.0 [2.0, 6.0]	−0.329 (−0.542, −0.116)	0.013

*Note:* Values are presented as median [interquartile range].

Abbreviations: APACHE, acute physiology and chronic health evaluation; BMI, body mass index; CCI, Charlson comorbidity index; CDR, clinical dementia rating; CRRT, continuous renal replacement therapy; IABP, intra‐aortic balloon pumping; ICDSC, intensive care delirium screening checklist; PCPS, percutaneous cardiopulmonary support.

^a^ Explanatory variables in propensity score analysis: age, sex, Brinkman index, BMI, CCI, CDR, APACHE II score, SOFA, emergency surgery, CRRT, IABP, PCPS and psychotropics for delirium or insomnia from ICU admission through the first outdoor session, and the highest ICDSC score.

^b^ Wilcoxon rank–sum test for continuous variables; effect size reported as Cliff's *δ* with 95% confidence intervals.

### 4.5 Dose–Response Analyses

In the adjusted linear regression (fitted after multiple imputation), a greater number of outdoor sessions was independently associated with a lower ICDSC score at ICU discharge (standardised *β* = −0.113; *p* = 0.025) (Table 3). In addition, the variance inflation factor was < 2 for all explanatory variables (Table S2).

**TABLE 3:** Association between the presence and number of outdoor therapy sessions and ICDSC score at ICU discharge.

	Simple regression analysis				Multiple regression analysis^a^				
	*B*	SE	*R* ^2^	*p*	*B*	*β*	SE	Adjusted *R* ^2^	*p*
Outdoor therapy (presence)	0.127	0.334	< 0.001	0.705	−0.651	−0.101	0.307	0.363	0.035
Outdoor therapy (number)	0.553	0.147	< 0.001	0.933	−0.362	−0.113	0.161	0.364	0.025

*Note:* *N* = 391, Values are presented as median [interquartile range].

Abbreviations: *B*, partial regression coefficient; *β*, standardised partial regression coefficient; APACHE, acute physiology and chronic health evaluation; BMI, body mass index; CCI, Charlson comorbidity index; CDR, clinical dementia rating; CRRT, continuous renal replacement therapy; IABP, intra‐aortic balloon pumping; ICDSC, intensive care delirium screening checklist; PCPS, percutaneous cardiopulmonary support.

^a^ Explanatory variables in multiple regression analysis: age, sex, Brinkman index, BMI, CCI, CDR, APACHE II score, SOFA, emergency surgery, CRRT, IABP, PCPS and psychotropics for delirium or insomnia from ICU admission through the first outdoor session, and the highest ICDSC score, highest ICDSC score.

## 5 Discussion

In this single‐centre retrospective cohort of adults with prolonged ICU stays, supervised outdoor exposure was associated with three convergent signals of its benefit on delirium severity: same‐day within‐patient reduction in ICDSC scores at the first session, lower ICDSC scores at ICU discharge in propensity‐score–matched comparisons, and an adjusted dose–response relationship in which additional sessions were correlated with lower discharge ICDSC scores. Importantly, these associations emerged despite the outdoor‐therapy group being older, exhibiting greater delirium burden during the ICU course and receiving psychotropic agents more frequently—features addressed through multiple imputation and rigorous matching.

Several design elements strengthen our inference. First, the immediate analysis used synchronised assessments (16:00 vs. 16:00) and verified medication stability in the preceding 72 h, limiting the impact of short‐term pharmacological confounding on the index exposure. Second, the integration of propensity methods with imputed datasets achieved balance across clinical severity, device support and pre‐session psychotropic exposure, thus mitigating confounding by indication. Third, outdoor sessions were implemented once the highest ICDSC score was recorded, reducing the likelihood that the observed improvements reflect simple regression to the mean or a threshold‐for‐eligibility effect.

These findings fit the established pathophysiology [8, 9, 10, 11, 22, 23, 25]. Naturalistic light, airflow and multisensory cues plausibly reinforce circadian entrainment, attenuate cognitive load and sensory mismatch, and support affective regulation. Moreover, patients in the ICU are routinely exposed to environmental stressors—alarms and device noise, sounds from neighbouring patients, constant illumination, sleep disruption and the use of physical restraints [39, 40, 41, 42]. The confined environment has been associated with adverse psychological and behavioural changes, including depressive symptoms, impaired sleep quality and increased problematic behaviours [43, 44, 45]. By replacing—even briefly—this high‐stress milieu with a more open, natural setting, supervised outdoor exposure may counter these delirium precipitants and improve vigilance, mood and rest. Operationally, escorted outdoor sessions function as a pragmatic extension of early mobilisation that can be layered onto existing non‐pharmacological bundles without introducing additional sedatives or restraints [46, 47]. While causality cannot be claimed owing to an observational design, the alignment of immediate change, between‐group difference and dose–response outcomes indicates internal coherence and a clinically meaningful observation, warranting prospective confirmation.

Finally, the feasibility of outdoor therapy across the modes of transportation—bed transport, wheelchair and limited ambulation—suggests that it can be integrated into multidisciplinary rounds with standard safety checks, including weather, staffing, monitoring requirements and device‐specific considerations (e.g., extracorporeal circuits and ventilator transitions). Programmatic documentation of dose elements (time of day, duration, frequency and ambient conditions) can enable learning‐health‐system cycles and future definitive trials.

## 6 Limitations

This study has several important limitations. First, its observational design leaves room for residual confounding despite multiple imputation and propensity‐score matching; unmeasured factors—such as the trajectory of agitation, family presence or staffing intensity—may have influenced both eligibility for outdoor therapy and subsequent delirium outcomes. Second, although psychotropic exposure up to the first outdoor session was modelled, and no initiation or discontinuation occurred within the preceding 72 h for the immediate analysis, we could not comprehensively capture post‐exposure medication dynamics—dose escalations or tapers, deprescribing of drug classes or cross‐titration—which may have biased the ICDSC scores at ICU discharge; the direction of this bias is uncertain, as de‐escalation might unmask delirium, whereas rationalisation could improve cognition independently of outdoor exposure. Third, key time‐dependent variables such as mechanically ventilated days and ICU length‐of‐stay days were observed after the initial outdoor exposure and lie on the causal pathway for many patients; to avoid adjustment‐for‐mediator bias, we did not include them as conventional covariates, but their omission means time‐dependent confounding may have persisted, suggesting that future work should consider marginal structural models or related g‐methods. Fourth, the ICDSC is a screening tool rather than a granular severity scale; although previous studies have used ICDSC scores to assess delirium severity [48, 49, 50], misclassification and ceiling or floor effects are possible. Fifth, the intervention dose was heterogeneous—the frequency, duration and environmental context were not protocolised—and long‐term cognitive or functional outcomes were not assessed. Finally, because outdoor sessions were delivered after initial stabilisation and after the peak ICDSC score was reported, selection and timing effects could have persisted even after matching. Taken together, these limitations warrant cautious interpretation as well as protocolised, prospective—ideally multicentre—studies that incorporate time‐aware causal methods, detailed medication tracking, and prespecified dosing parameters.

## 7 Implications for Practice and Research

Escorted outdoor exposure appears implementable across device supports when embedded in multidisciplinary workflows. Such programmes should (i) formalise safety screening (physiological stability, device checklists and weather/air quality thresholds), (ii) standardise dose capture (clock time, duration, frequency and illuminance/lux if feasible), (iii) document concurrent non‐pharmacological bundle elements and (iv) track psychotropic trajectories before and after the sessions. Future trials should randomise or sequence outdoor exposure within a protocolised mobilisation bundle, stratified by delirium risk and ventilatory status, and evaluate patient‐centred outcomes including delirium‐free/coma‐free days, sleep quality and post‐ICU cognition.

## 8 Conclusions

In this single‐centre, retrospective cohort study of adults with ICU stays ≥ 7 days, we evaluated whether supervised outdoor therapy was associated with reduced delirium severity. Outdoor therapy was associated with same‐day improvement at first exposure and with lower ICDSC scores at ICU discharge in propensity‐score–matched comparisons, with a negative dose–response after adjustment. These findings support the integration of supervised outdoor exposure as a pragmatic, non‐pharmacological adjunct to multimodal delirium care and justify protocolised prospective—ideally multicentre—trials to define standardised dosing, candidate selection and patient‐centred outcomes.

## Ethics Statement

This study was approved by the Ethics Committee of Shinshu University Hospital (approval number: 5624, approved on 14 September 2022).

## Consent

Informed consent was obtained from all patients or their families in accordance with institutional requirements.

## Conflicts of Interest

The authors declare no conflicts of interest.

## Supporting information


**Table S1:** Data on the status of outdoor therapy.
**Table S2:** Variance inflation factor for multiple regression analysis.
**Figure S1:** Representative outdoor‐therapy sessions across acuity levels and device support.
**Figure S2:** Flow chart showing the inclusion and exclusion process.
**Figure S3:** Distribution of missing values.
**Figure S4:** Distribution of propensity scores before and after matching.
**Figure S5:** Standardised mean difference of propensity scores before and after matching.

## Data Availability

The data that support the findings of this study are available on request from the corresponding author. The data are not publicly available due to privacy or ethical restrictions.
