# Supplementary material for: Effects of Outdoor Therapy on Delirium in Patients With Prolonged Intensive Care Unit Stays: A Single‐Centre Retrospective Study
Source: Nurs Crit Care. 2025 Nov 19;30(6):e70263. doi: 10.1111/nicc.70263 (PMC12628662; doi:10.1111/nicc.70263)
Supplement: Supplementary file 1 — Table S1: Data on the status of outdoor therapy. Table S2: Variance inflation factor for multiple regression analysis. Figure S1: Representative outdoor‐therapy sessions across acuity levels and device support. Figure S2: Flow chart showing the inclusion and exclusion process. Figure S3: Distribution of missing values. Figure S4: Distribution of propensity scores before and after matching. Figure S5: Standardised mean difference of propensity scores before and after matching. [file NICC-30-0-s001.docx]

**Table S1. Data on the status of outdoor therapy**

|  | Outdoor therapy group  n = 59 |
| --- | --- |
| Days to First Outdoor Therapy Session — day | 22.0 [15.0, 43.0] |
| number of sessions — no. (%) |  |
| 1 session | 41 (69.4) |
| 2 sessions | 11 (18.6) |
| 3 sessions | 1 (1.6) |
| 4 sessions | 5 (8.4) |
| 5 sessions | 2 (3.3) |
| Mode of transportation — no. (%) |  |
| Bed | 8 (13.5) |
| Wheelchair | 49 (83.0) |
| Walking | 2 (3.3) |

Values are presented as median [interquartile range] or no. (%)

**Table S2. Variance inflation factor (VIF) for multiple regression analysis**

|  |  |
| --- | --- |
| Explanatory variable | VIF |
| Outdoor therapy (number) | 1.566529 |
| Age | 1.666530 |
| Sex | 1.268075 |
| BMI | 1.117480 |
| Brinkman index | 1.331469 |
| CCI | 1.618733 |
| CDR | 1.125542 |
| Emergency surgery | 1.292435 |
| APACHE II score | 1.856595 |
| SOFA | 1.997983 |
| CRRT | 1.772951 |
| IABP | 1.198486 |
| PCPS | 1.872768 |
| Highest ICDSC score | 1.393497 |
| Medications for ICU delirium |  |
| Aripiprazole | 1.132366 |
| Asenapine | 1.171732 |
| Olanzapine | 1.143146 |
| Perospirone | 1.211598 |
| Quetiapine | 1.189422 |
| Risperidone | 1.232059 |
| Milnacipran | 1.064787 |
| Mirtazapine | 1.087756 |
| Trazodone | 1.179640 |
| BZRA/anxiolytics | 1.186967 |
| BZRA/hypnotic | 1.141360 |
| Non-BZRA/hypnotic | 1.214702 |
| Lemborexant | 1.492548 |
| Ramelteon | 1.425259 |
| Suvorexant | 1.210262 |
|  |  |

Abbreviations: APACHE, acute physiology and chronic health evaluation; BMI, body mass index; BZRA, benzodiazepine receptor agonists; CCI, Charlson comorbidity index; CDR, clinical dementia rating; CRRT, continuous renal replacement therapy; IABP, intra-aortic balloon pumping; ICDSC, intensive care delirium screening checklist; PCPS, percutaneous cardiopulmonary support

**Figure S1. Representative outdoor-therapy sessions across acuity levels and device support**

Composite of four supervised outdoor sessions delivered to critically ill adults, demonstrating feasibility across mobility and support needs. All sessions were conducted under multidisciplinary oversight (nurses, physicians, and physical/occupational therapists); a clinical engineer attended when extracorporeal circuits were present. Faces are blurred to protect privacy.

(A) Bed transport outdoors beneath spring cherry blossoms; patient remained bedbound with continuous monitoring and multiple infusions.

(B) Wheelchair session during central extracorporeal membrane oxygenation (ECMO); lines and circuit secured, with clothing adjusted for cold weather.

(C) Second wheelchair session on central ECMO, illustrating delivery in a patient with high physiological dependency under close supervision.

(D) Seated session on a transportable ICU bed after transition to a portable ventilator; devices consolidated on a mobile rack and airway support maintained.

Abbreviations: ECMO, extracorporeal membrane oxygenation; ICU, intensive care unit; MCS, mechanical circulatory support


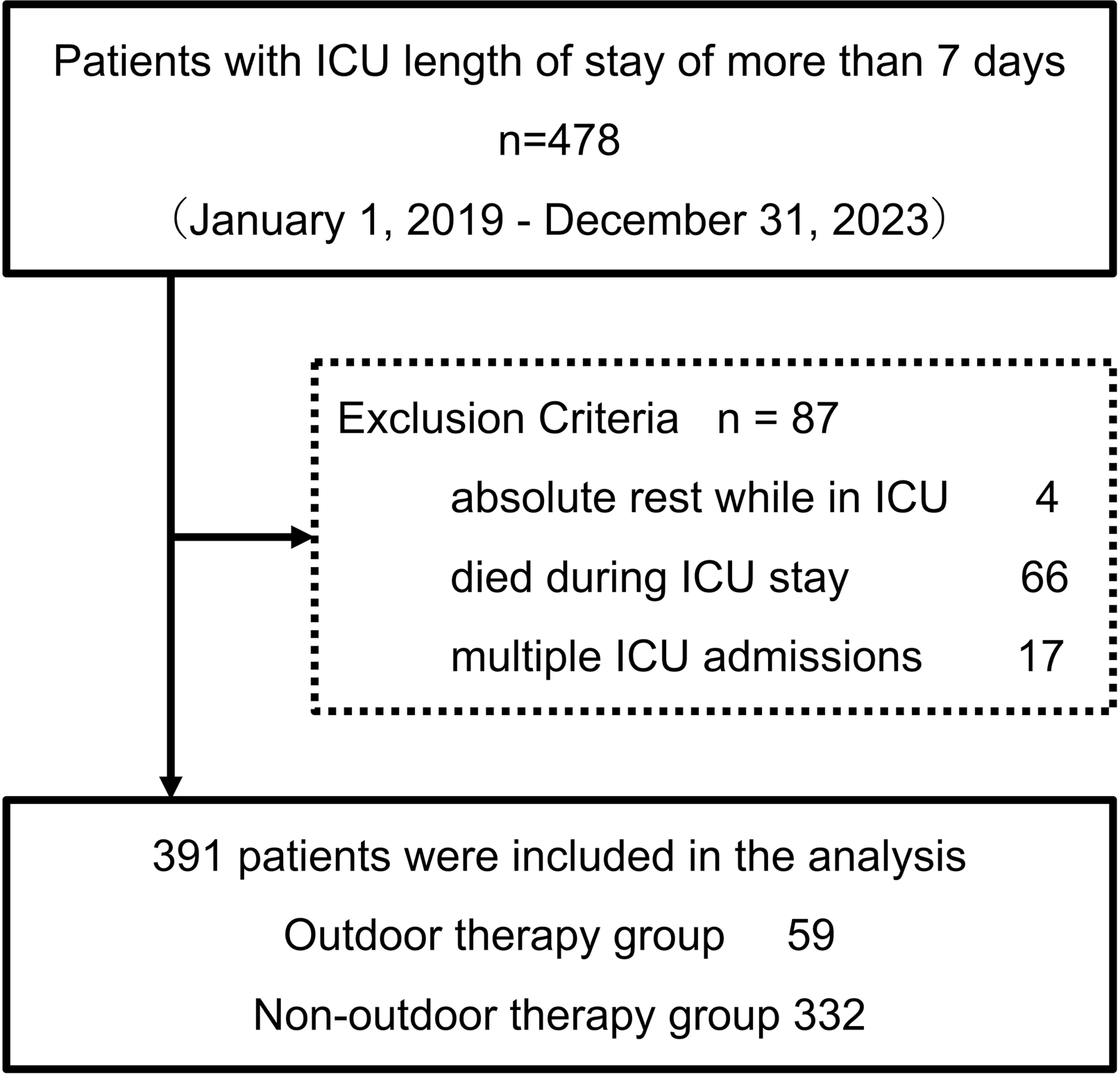


**Figure S2. Flow chart showing the inclusion and exclusion process**


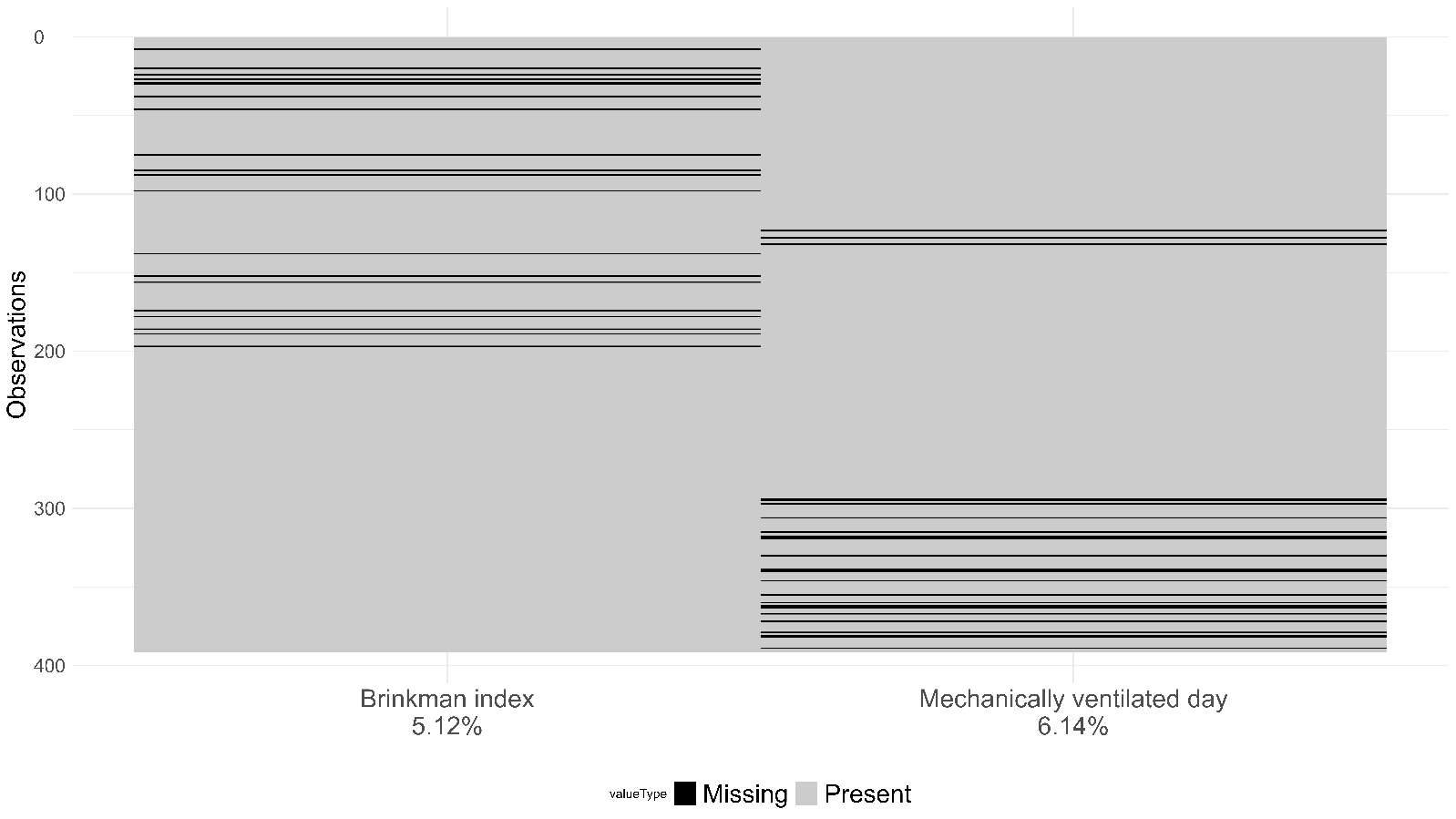


**Figure S3. Distribution of missing values**


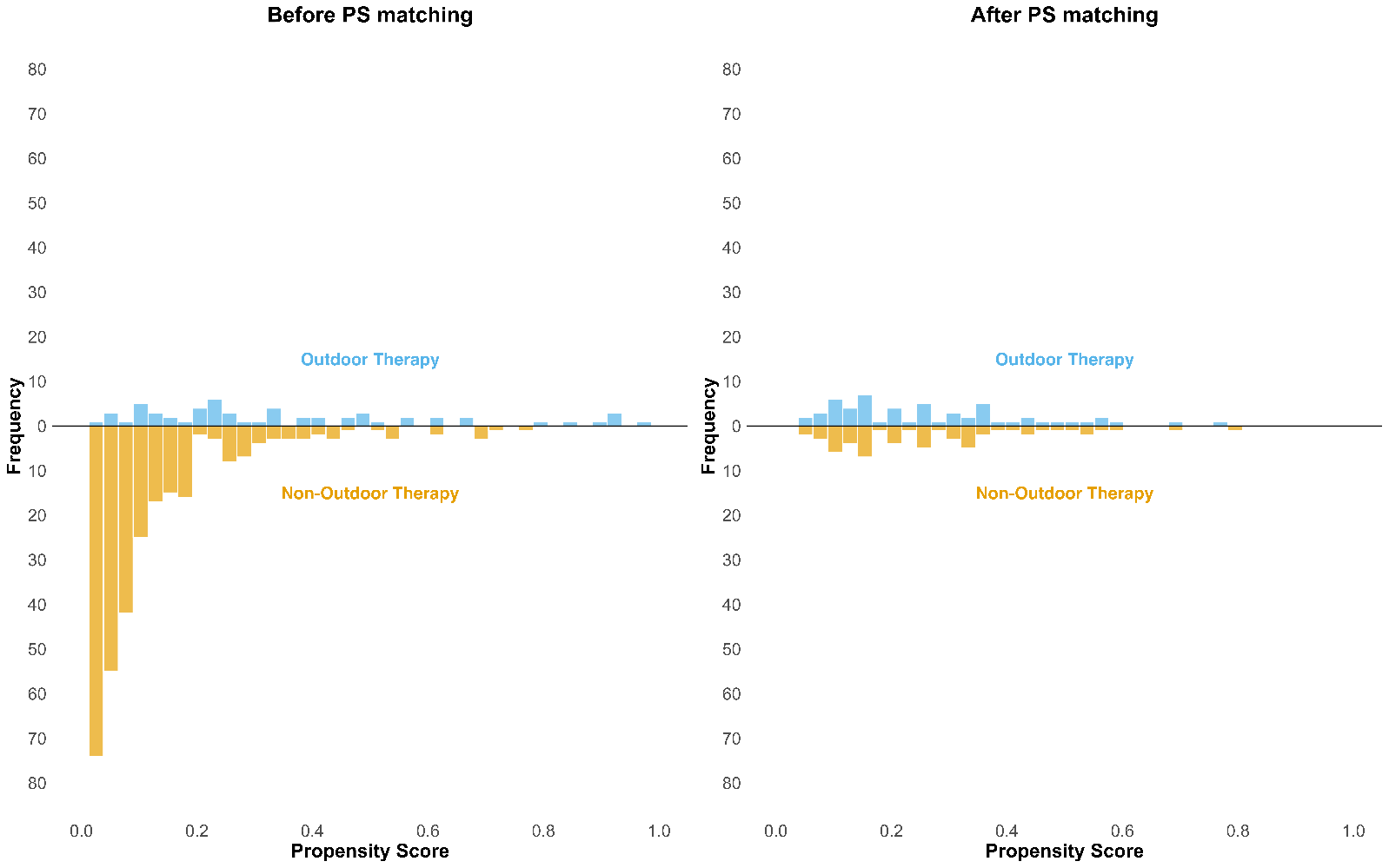


**Figure S4. Distribution of propensity scores before and after matching**


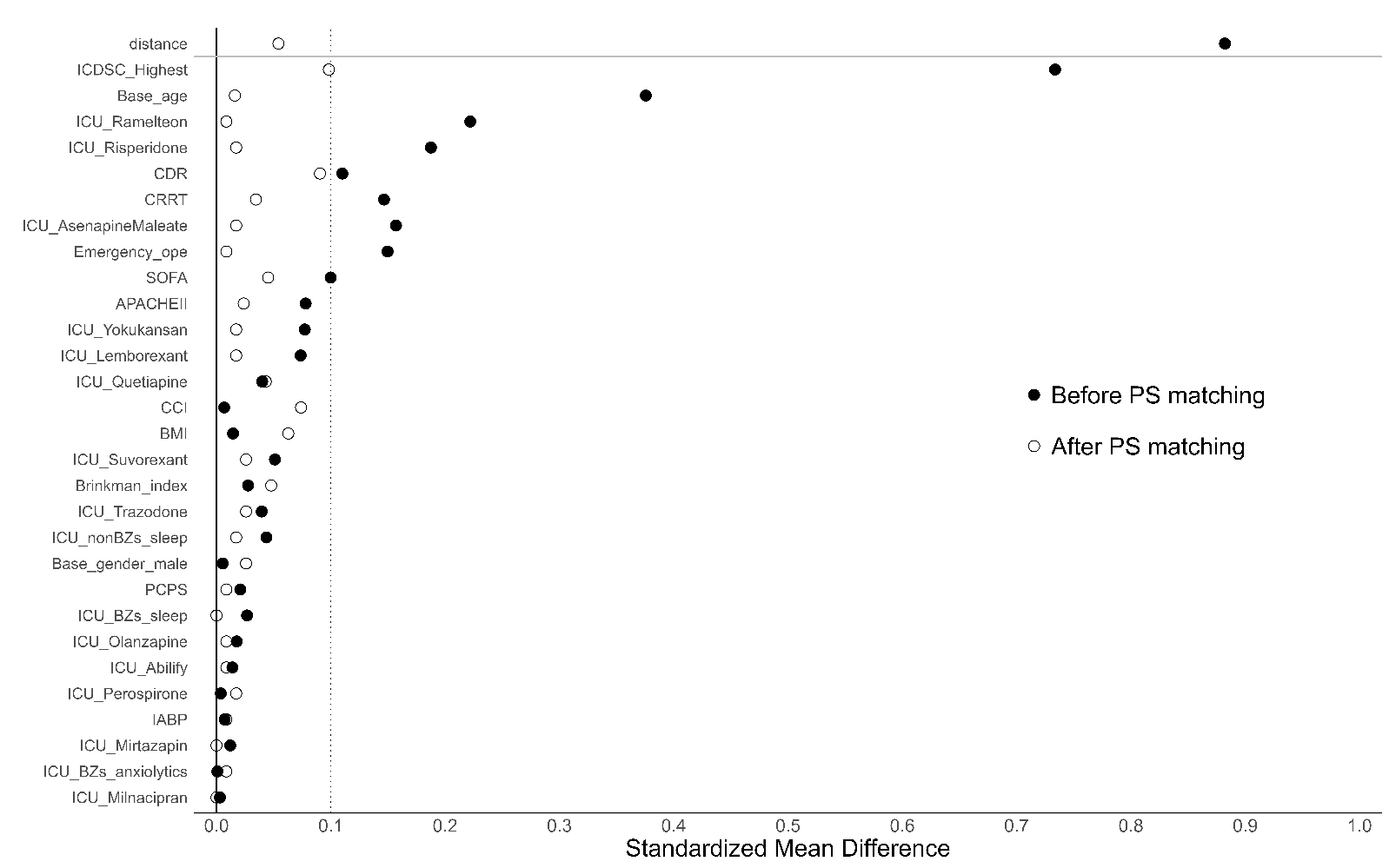


**Figure S5. Standardized mean difference of propensity scores before and after matching**

Abbreviations: PS, Propensity Score
